# Performance evaluation of dextran-coated CaFe_12_O_19_/MnFe_2_O_4_ exchange-spring composites for the self-heating properties at radio frequency field

**DOI:** 10.3389/fchem.2024.1347113

**Published:** 2024-03-05

**Authors:** Ishtiaque Syed, Sharmin Akter Lima, Nandita Deb, M. Al-mamun, Sheikh Manjura Hoque

**Affiliations:** ^1^ Centre for Advanced Research in Sciences (CARS), University of Dhaka, Dhaka, Bangladesh; ^2^ Department of Physics, University of Dhaka, Dhaka, Bangladesh; ^3^ Materials Science Division, Atomic Energy Centre, Dhaka, Bangladesh

**Keywords:** composite, magnetic properties, exchange-spring coupling, specific loss power (SLP), hyperthermia

## Abstract

The CaFe_12_O_19_/MnFe_2_O_4_ composites with the hard (CaFe_12_O_19_) and soft (MnFe_2_O_4_) magnetic phases, were prepared by chemical co-precipitation method. The prepared composites were calcined at three different temperatures to form different phases. The structural, morphological, and magnetic properties of composite were analyzed by X-ray diffraction (XRD), Fourier transform infrared spectroscopy (FTIR), room temperature vibrational sample magnetometer (VSM), and transmission electron microscopy (TEM). The presence of the hard and soft phases has been confirmed without any secondary phase from XRD analysis, indicating the formation of composite. The crystallite size is found to be in the range of 24–44 nm calculated by Scherrer’s formula. The TEM revealed hexagonal platelets of CaFe_12_O_19_ with spinel MnFe_2_O_4_ particles with an average particle size of 48 nm formed at the surface of the CaFe_12_O_19_/MnFe_2_O_4_ composite. The room temperature magnetic properties of composite were evaluated by employing VSM. The magnetic measurements have displayed enhancement in coercivity and magnetization for CaFe_12_O_19_/MnFe_2_O_4_, indicating that the composite possessed excellent exchange coupling. The composite’s enhanced energy product ((BH)_max_) made it highly promising for biomedical applications such as hyperthermia. The exchange-spring coupled magnetic composite was coated with dextran to make it biocompatible, which is necessary for hyperthermia applications. The coating was confirmed using Fourier transform infrared spectroscopy (FTIR). Cytotoxicity tests on Vero cell lines showed that the coated composites had an excellent (>95%) cell survival rate. The hyperthermia heating of composite was measured for different concentrations of composite (0.25, 0.5, 1, 2, and 4 mg/mL) from which specific loss power (SLP) was calculated. From these SLP values, the optimized concentration was identified.

## 1 Introduction

The exchange-spring mechanism is a process by which the magnetic properties of soft and hard ferrites can be coupled together. It is a type of magnetic coupling that occurs between two magnetic materials with different coercivities. The soft magnetic material, which has a low coercivity, is coupled to the hard magnetic material, which has a high coercivity. This coupling is caused by the exchange interaction, which is a quantum mechanical effect that occurs between neighboring magnetic moments. This is achieved by creating a composite material that contains both soft and hard ferrites (Skomski and Coey, 1994; Hoque et al., 2013; Remya et al., 2016; Song et al., 2011). In the early 1990s, Kneller and Hawig proposed the theory of the exchange coupling concept between hard and soft phases (Kneller and Khan., 1987).

Exchange-spring magnetic composites are a type of magnetic composite that is particularly well-suited for magnetic particle hyperthermia (Lee et al., 2011). These nanoparticles consist of two different magnetic phases. In an exchange-spring system, two magnetic phases are coupled together, such that the magnetization of the soft phase is “pinned” to the magnetization of the hard phase. This means that when an alternating magnetic field is applied to exchange-spring magnetic nanoparticles, the two magnetic phases try to align with the field.

However, the exchange interactions prevent them from fully aligning. This creates a state of stress in the composites, which leads to the generation of heat (Peiravi et al., 2022).

Exchange-spring composites are typically made of a hard magnetic core and a soft magnetic shell. The hard core provides a high coercive field, which helps to keep the soft shell aligned with the applied magnetic field. The soft shell has a higher saturation magnetization than the hardcore, which means that it can generate more heat when it is rotated by the applied field (Roy and Kumar, 2009).

Exchange-spring magnetic composites have been shown to be more effective at generating heat than single-phase magnetic particles. This is because the exchange-spring coupling helps to prevent the soft phase from demagnetizing, which would otherwise reduce the amount of heat that is generated. The heat generated by exchange-spring magnetic particles is more localized than the heat generated by other types of magnetic particles. This is because the exchange interactions between the two magnetic phases help to prevent the heat from spreading. This makes exchange-spring magnetic particles more suitable for treating tumors that are close to sensitive tissues, such as the brain, spinal cord, eyes, blood vessels, *etc.* (Lee et al., 2011).

Calcium and manganese are two very essential body elements that play an important role in bodily functions. Moreover, they are non-toxic. The composites made by with those two ferrites will be highly efficient for hyperthermia application and their intake rate would be minimum.

## 2 Experimentation

CaFe_12_O_19_/MnFe_2_O_4_ composite ferrites were generated in this study using the chemical co-precipitation process. The chemical co-precipitation method is a simple, versatile, and scalable method for synthesizing CaFe_12_O_19_/MnFe_2_O_4_ composite. Again, the method is relatively inexpensive and does not require the use of toxic chemicals (Amighian et al., 2006; Pullar, 2012). This process was used to produce the composites of hard and soft ferrites in a 1:3 weight ratio. To produce the CaFe_12_O_19_/MnFe_2_O_4_ composite, CaCl_2_.2H_2_O, MnCl_2_.4H_2_O, FeCl_3_ salts were dissolved in deionized water under vigorous stirring at 95°C. After 8M NaOH solution (excess base concentration) was added to the solution, the pH of the solution was maintained at 11. The solution’s color swiftly changed from brown to black. The composite was filtered and extensively washed with deionized water to remove chloride ions, followed by multiple washes with ethanol to remove any remaining unwanted salts, and lastly dried in a vacuum at 90°C for 72 h. The sample was then ground in a mortar to separate the agglomerated particles to get fine powder which was in an amorphous state. The fine powder was first pelletized, and then it was calcined at three different temperatures (600°C, 800°C, 1,000°C). After the calcination process, the final product was ready, and in this product, all the phases of the composite were formed. CaFe_12_O_19_/MnFe_2_O_4_ composite were coated with dextran for magnetic particle hyperthermia. After several steps, dextran coated CaFe_12_O_19_/MnFe_2_O_4_ composite samples with a concentration of 20 mg/mL were prepared. The concentration of the prepared samples was diluted to three different concentrations of 2 mg/mL, 1 mg/mL, and 0.5 mg/mL for hyperthermia application.

## 3 Results and discussions

Qualitative X-ray diffraction analysis is a technique that uses X-rays to identify the crystalline phases present in a material. X-ray diffraction technique has been utilized to determine the structure, crystallite size, and lattice parameter of prepared CaFe_12_O_19_/MnFe_2_O_4_ composite ferrites. In this composite, hard ferrite and soft ferrite are mixed in 1–3 ratio.

CaFe_12_O_19_:MnFe_2_O_4_ = 1:3.

**FIGURE 1:**
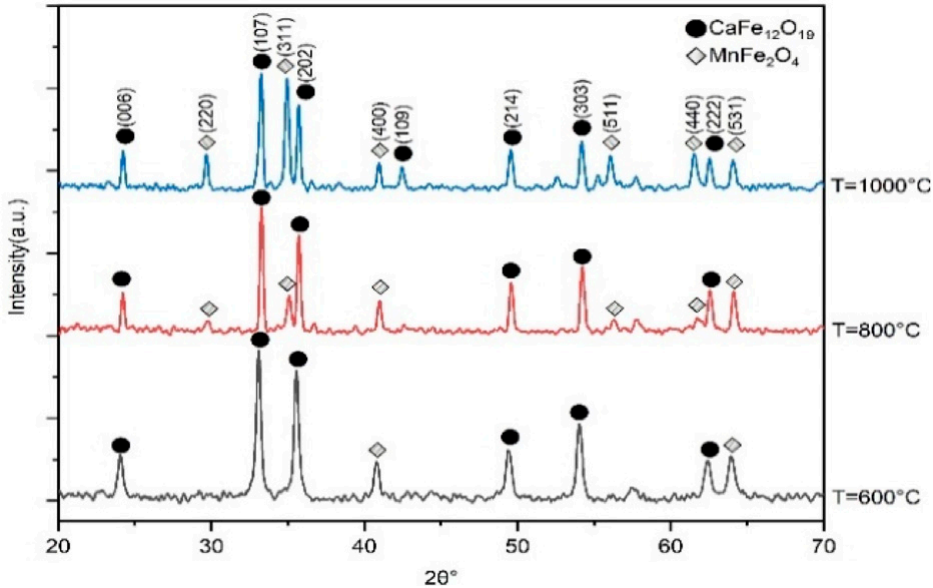
X-ray diffraction pattern of CaFe_12_O_19_/MnFe_2_O_4_ composite ferrites at three different calcination temperatures (600°C, 800°C, 1,000°C).

The XRD pattern in Figure 1 shows the mixed ferrite phases of the CaFe_12_O_19_/MnFe_2_O_4_ composite ferrites. Phase formation usually is identified by the comparison of peaks with standard JCPDS card (card no. 00-049-1,586 and card no.01-073–1964) reference data values. The highest intensity diffraction peak of all the samples was found at (107) orientation. The XRD pattern consists of standard reflecting planes (006), (107), (202), (109), (214), (303), and (222) confirming that the prepared samples belong to M-type hexaferrite, and the structure of the crystal is a hexagonal close-packed structure. Again, it is also clear from the phase identification that the phase is indeed of the crystalline MnFe_2_O_4_ particles, and the crystal structure is cubic spinel structure.

The lattice parameter of manganese ferrite (soft ferrite), which has a cubic spinel structure, was determined using the formula (Dhiman et al., 2008),

$$a=d\sqrt{h^{2}+k^{2}+l^{2}}$$

Where, the lattice parameter is denoted by a, interplanar spacing is denoted by d, and h, k, and l are the miller indices for the respective planes. The lattice constants (a and c) and lattice Volume of unit cell (V_cell_) of calcium hexaferrite (hard ferrite), which has a hexagonal close packed (hcp) structure, were calculated by using following equations (Shinde et al., 2020),

$$\frac{1}{d^{2}}=\frac{4}{3}\left(\frac{h^{2}+hk+k^{2}}{a^{2}}\right)+\frac{l^{2}}{c^{2}}$$

$$V_{\text{cell}}=\frac{\sqrt{3}}{2}a^{2}c$$

The lattice parameter for manganese ferrite (MnFe_2_O_4_) is 8.590Å. The c/a ratio of 3.667, which is slightly below the standard value of 3.77, confirms the hexagonal close-packed crystal structure of calcium hexaferrite.

**FIGURE 2:**
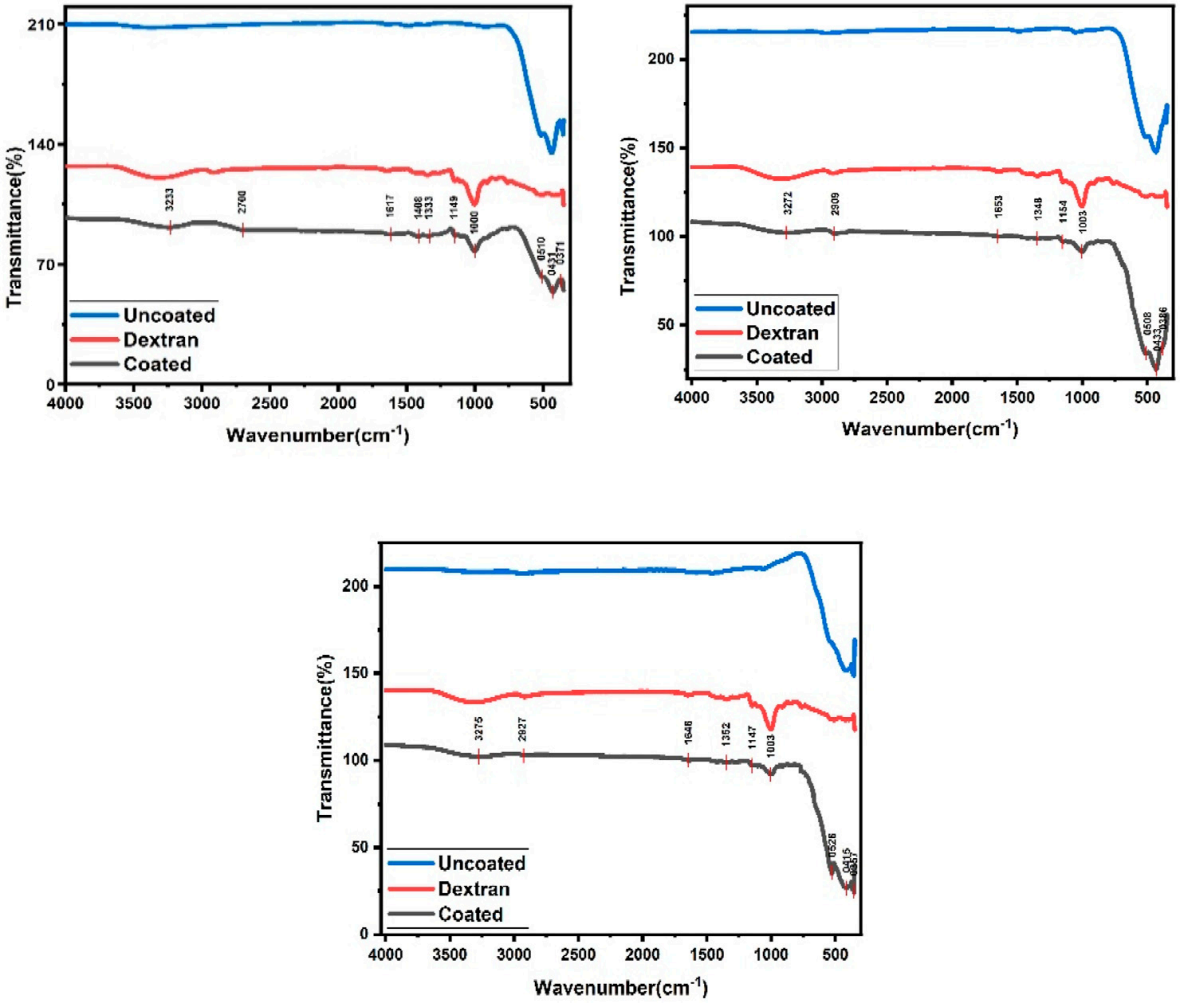
FTIR spectra for CaFe_12_O_19_/MnFe_2_O_4_ nanocomposite sample when the calcination temperatures are respectively 600°C, 800°C, and 1,000°C.

FTIR spectrum is a measurement which can detect whether a specific bond with a certain bond strength is present in a compound. In this study, FTIR analysis was performed to confirm successful dextran coating on CaFe_12_O_19_/MnFe_2_O_4_ composite ferrites. Possible interactions between composites calcined at three temperatures (600°C, 800°C, and 1,000°C) were analyzed. FTIR spectra for bare CaFe_12_O_19_/MnFe_2_O_4_ composite ferrites, dextran solution, dextran-coated sample solutions are depicted in Figure 2.

In Figure 2, FTIR spectrum of the bare composites exhibits the peak at 300–400 cm^-1^ which corresponds to the stretching vibrations of intrinsic metal oxygen at octahedral sites while the peak at 580–440 cm^-1^ represents the stretching vibrations of metal oxides at the tetrahedral and octahedral positions (Islam, K et al., 2020). Stretching peak at 541 cm^-1^ and 474 cm^-1^ indicates existence of metal-oxygen vibration mode of hexaferrite structure including octahedral and tetrahedral sites respectively. For dextran and coated composites, broad absorption peaks appeared at about 3,275 cm^-1^ which can be related to the presence of abundant hydroxyl (-OH) groups (Predescu et al., 2018). Comparing spectra for bare and dextran-coated composites, the appearance of some new absorption bands can be observed. For instance, the bands at about 1,147 cm^-1^ are due to the stretching vibration of C-N, and the band at 1,352 cm-1 is attributed to the bending vibration of the C–H bond. These data prove that the surface of magnetic composites has been covered with dextran polymer. It is believed that different interactions such as van der Waals force, hydrogen bond, and electrostatic interactions keep dextran on the surface of composites.

A physical property measurement system (PPMS) was used to investigate the magnetic properties of synthesized CaFe_12_O_19_/MnFe_2_O_4_ composite ferrites.

In this study, dextran-coated CaFe_12_O_19_/MnFe_2_O_4_ composite ferrites calcined at three different temperatures (600°C, 800°C, 1,000°C) were studied as magnetic hyperthermia agents. The amount of heat generated in magnetic hyperthermia depends on the heating potential, so magnetic characterization schemes were performed to determine the crucial factors for optimizing the heating potential.

Soft ferrites have high magnetic saturation (M_S_), high magnetic retentivity with low coercivity resulting in easy magnetization and demagnetization. Hard ferrites have high magnetic saturation with very high coercivity making them difficult to demagnetize and magnetize. The prepared CaFe_12_O_19_/MnFe_2_O_4_ composite shows exchange-spring coupling behavior, as evidenced by its saturation magnetization (M_S_) and remanent magnetization (M_R_) values that are higher than CaFe_12_O_19_ but lower than MnFe_2_O_4_. The coercivity (H_C_) of the prepared sample is also higher than MnFe_2_O_4_ but lower than CaFe_12_O_19_. Exchange-spring coupling in magnetic materials can increase saturation magnetization and remanent magnetization, which in turn decreases coercivity and increases magnetic energy product (Pahwa et al., 2017). In an exchange-spring magnet, the soft and hard magnetic phases are mixed on a nanometer scale. This allows the magnetic moments of the two phases to couple together, resulting in a material with high saturation magnetization, remanent magnetization, and coercivity (Ye et al., 2020). So, the prepared CaFe_12_O_19_/MnFe_2_O_4_ composite, which behaves like an exchange-spring coupling material, is highly applicable for hyperthermia application.

**FIGURE 3:**
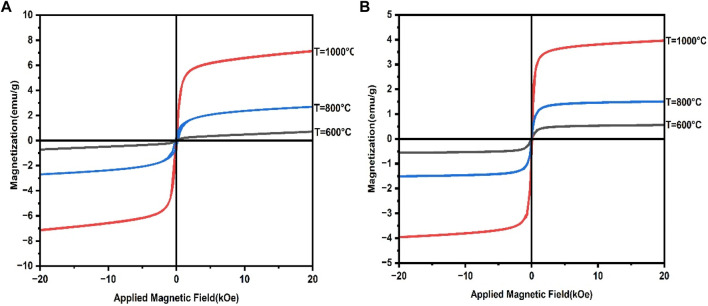
**(A)** M-H loop for uncoated CaFe_12_O_19_/MnFe_2_O_4_ composites at three calcination temperatures (600°C, 800°C, 1,000°C), **(B)** M-H loop for dextran-coated CaFe_12_O_19_/MnFe_2_O_4_ composites at three calcination temperatures (600°C, 800°C, 1,000°C).

Figure 3 (b) shows the M-H loop for dextran-coated CaFe_12_O_19_/MnFe_2_O_4_ composite samples calcined at three different temperatures. The hysteresis loops of the uncoated and coated composites ferrites are similar in shape, but the coated composites have a smaller coercivity and a lower magnetic saturation (M_S_). The smaller coercivity is due to the dextran coating, which provides a barrier to the movement of domain walls. This makes it more difficult to magnetize the composites, and hence the coercivity is reduced (Shaterabadi et al., 2017).

The lower M_S_ is due to the dextran coating, which interacts with the surface of the magnetite composites and disrupts the magnetic order. This reduces the overall magnetization of the composites (Shaterabadi et al., 2017). The remanent magnetization (M_R_) of the dextran-coated composites is nearly zero, indicating the superparmagnetic behavior of these samples (Shaterabadi et al., 2017). So, the prepared dextran-coated CaFe_12_O_19_/MnFe_2_O_4_ composites are superparamagnetic in behavior. The magnetic properties of the coated and uncoated samples changed in a similar way, although the values for the coated samples were slightly lower due to the coating.

**FIGURE 4:**
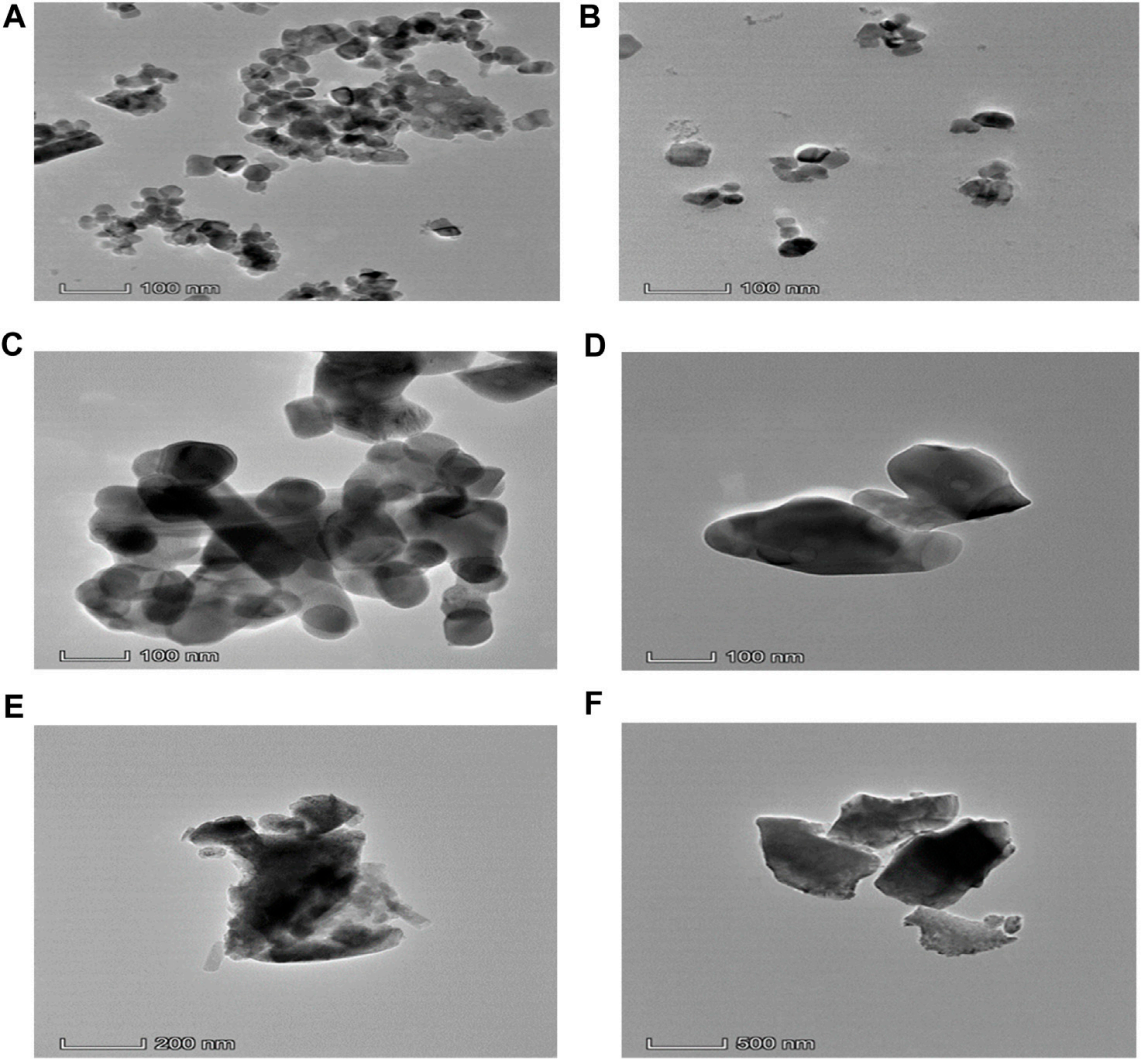
**(A)** TEM micrograph of uncoated composite calcined at 600°C, **(B)** TEM micrograph of dextran-coated composite calcined at 600°C, **(C)** TEM micrograph of uncoated composite calcined at 800°C, **(D)** TEM micrograph of dextran-coated composite calcined at 800°C, **(E)** TEM micrograph of uncoated composite calcined at 1,000°C, **(F)** TEM micrograph of dextran-coated composite calcined at 1,000°C.

Transmission Electron Microscopy (TEM) is an extremely strong technique for examining the particle shape. The TEM image clearly shows the crystal size and shape. TEM bright field images of uncoated and coated samples of CaFe_12_O_19_/MnFe_2_O_4_ composites calcined at three different temperatures (600°C, 800°C, 1,000°C). In the uncoated samples, the particles are aggregated; however, the dextran coating in the coated samples prevents aggregation and results in a dispersed sample (Figure 4). The average particle size was found to be greater due to the coating. The result from TEM is inconsistent with the result from XRD because XRD usually measures the average particle size, while TEM images can show individual, larger particles. From the TEM image, it can be seen that uniform distribution of hard and soft phases, which facilitates the exchange coupling between them. The hexagonal close-packed structure of the calcium hexaferrite (CaFe_12_O_19_) and cubic spinel structure of manganese ferrite (MnFe_2_O_4_) are confirmed by TEM image.

The different rings in the selected area diffraction pattern (SAED) correspond to different crystal planes that are present in the sample. From the SAED pattern, it can be seen that uniform distribution of hard and soft phases, which facilitates the exchange coupling between them. The hexagonal close-packed structure of the calcium hexaferrite (CaFe_12_O_19_) and cubic spinel structure of manganese ferrite (MnFe_2_O_4_) are confirmed by TEM images. The SAED pattern of nanocomposite ferrite confirms the presence of both the hard and soft phases. The coexistence of both the hard and soft phases was confirmed by selected area electron diffraction (SAED) pattern of (006) (107), (202) (303) planes of calcium hexaferrite (CaFe_12_O_19_) and (311) (511), (440) of planes of manganese ferrite (MnFe_2_O_4_).

**FIGURE 5:**
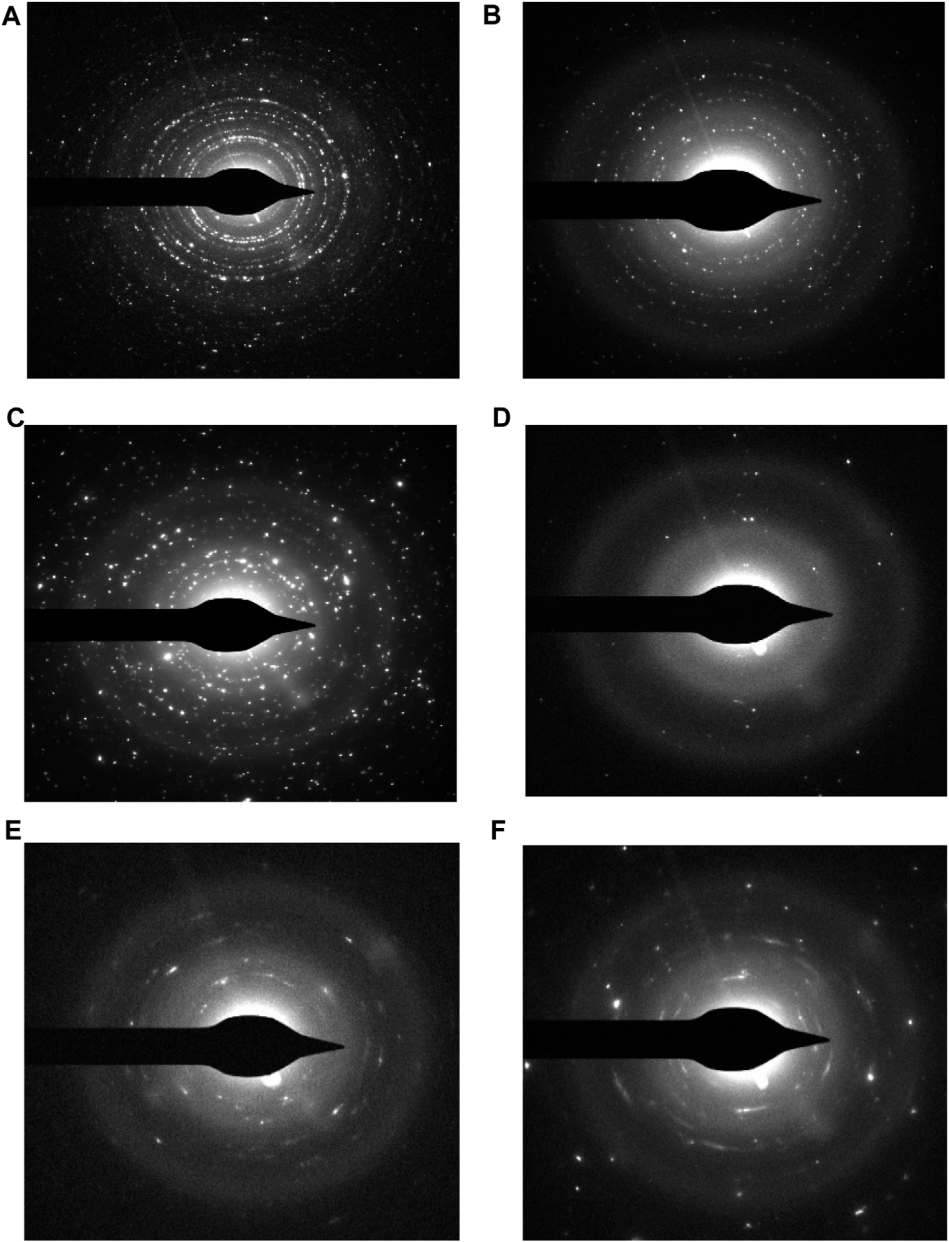
Selected area diffraction pattern for- **(A)** uncoated composite at calcination temperature 600°C, **(B)** dextran-coated composite at calcination temperature 600°C, **(C)** uncoated composite at calcination temperature 800°C, **(D)** dextran-coated composite at calcination temperature 800°C, **(E)** uncoated composite at calcination temperature 1,000°C, **(F)** dextran-coated composite at calcination temperature 1,000°C.

SAED is a powerful tool for characterizing the crystal structure of materials (Tivol, 2010). It can be used to identify the crystal structure of materials. The series of rings in Figure 5 confirms the polycrystallinity of the prepared CaFe_12_O_19_/MnFe_2_O_4_ composite.

**FIGURE 6:**
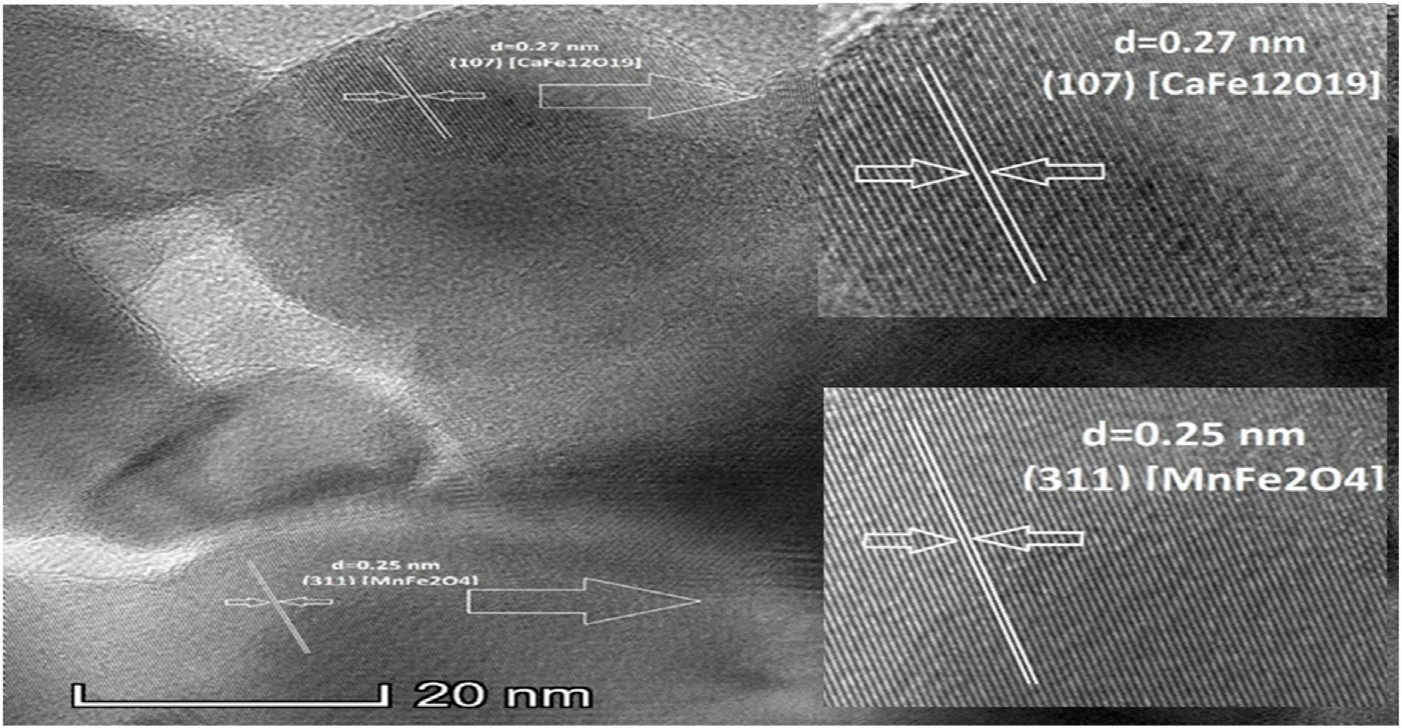
High resolution TEM (HRTEM) image for uncoated composite when calcination temperature 1,000°C.

Figure 6 provides the details of the interplanar distance of hard magnetic phase 0.27nm, which belongs to CaFe_12_O_19_ (107) crystallographic plane (Shinde and Dahotre, 2021) and soft magnetic 0.25 nm magnetic phases belongs to MnFe_2_O_4_ (311) crystallographic plane (Zipare et al., 2015). The lattice fringes of both hard and soft magnetic phases are well matched with the theoretical values from P63/mmc and Fd-3m for the calcium hexaferrite (CaFe_12_O_19_) and manganese ferrite (MnFe_2_O_4_) phases, respectively.

**FIGURE 7:**
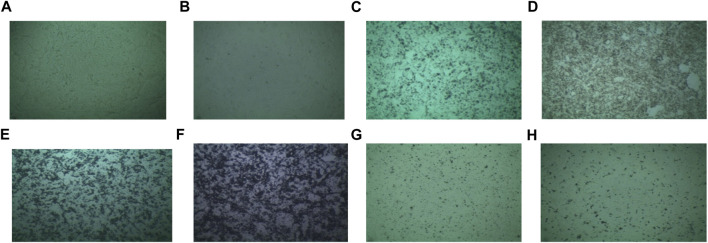
Medium (Vero) **(A)** sample without solvent, **(B)** sample with solvent, **(C)** 2 mg/mL concentrated uncoated sample calcined at 600°C, **(D)** 2 mg/mL concentrated coated sample calcined at 600°C, **(E)** 2 mg/mL concentrated uncoated sample calcined at 800°C, **(F)** 2 mg/mL concentrated coated sample calcined at 800°C, **(G)** 2 mg/mL concentrated uncoated sample calcined at 1,000°C, **(H)** 2 mg/mL concentrated coated sample calcined at 1,000°C.

For employing any sample in a biomedical experiment, it is essential to know whether it is biocompatible or not. In this study, the cytotoxicity of dextran-coated CaFe_12_O_19_/MnFe_2_O_4_ composite ferrites was evaluated on the Vero cell line, a kidney epithelial cell extracted from an African green monkey (Figure 7). For this test, samples having a concentration of 2 mg/mL were provided.

**FIGURE 8:**
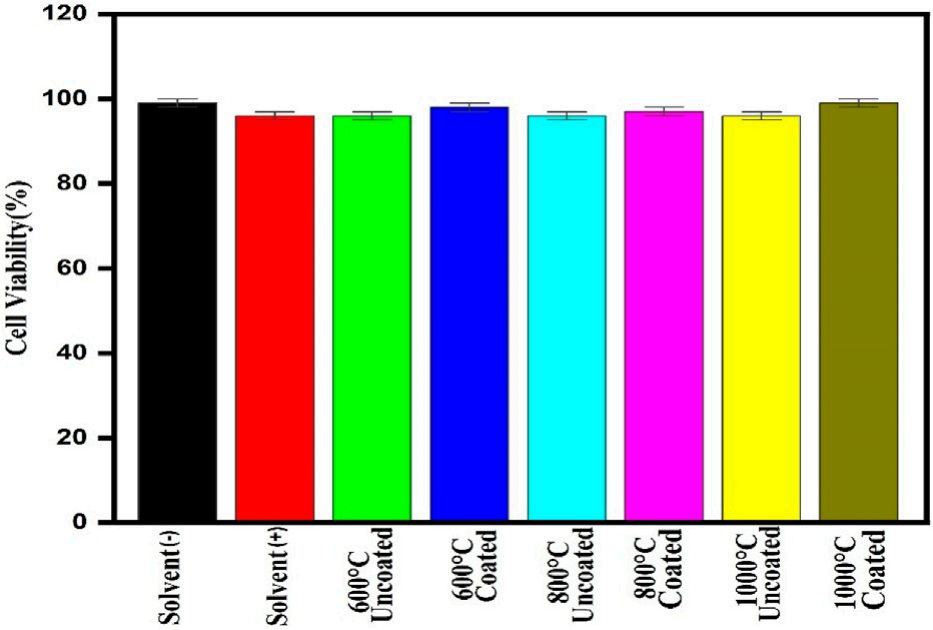
Cell survival rate of the prepared composite at 2 mg/mL concentrations on Vero cell lines.

The survival of the cell is more than 95% for Vero cell lines which can be said to be nontoxic easily (Figure 8). So, the prepared dextran-coated CaFe_12_O_19_/MnFe_2_O_4_ composites are nontoxic.

Magnetic composites suspended in a liquid medium (ferrofluid) can create heat due to magnetic losses when exposed to a high frequency alternating magnetic field. Localized heat can cause cancer cells to die. Composites that can increase temperature up to 46°C are suitable for cancer treatment. Faster treatment with a low metal content is highly desirable for hyperthermia applications (Li et al., 2013; Yu et al., 2021). Furthermore, for effective therapy, the temperature of cancerous tissue needs to reach 42°C − 46°C (Kim et al., 1982; Garcia et al., 2012; Wolf., 2008).

Magnetic composites should be synthesized with the following restrictions in mind for hyperthermia application: To begin, they should have the maximum feasible specific loss power (SLP) within the field and frequency range considered safe for the human body in order to minimize any adverse effects and to be beneficial for treating tiny tumors [Dutz and Hergt (2014)], second, they should be near superparamagnetic with minimal magnetostatic interactions in order to minimize aggregating, and third, they should be biocompatible with moderate cytotoxicity.

Hyperthermia data analysis is used to measure the heating properties of dextran-coated CaFe_12_O_19_/MnFe_2_O_4_ nanocomposite ferrites with varying concentrations (0.25 mg/mL, 0.5 mg/mL, 1 mg/mL, 2 mg/mL, and 4 mg/mL) to determine the relation between time and temperature. The heating property of prepared nanoparticles upon using an AC magnetic field with frequency 327 kHz and the amplitude of the applied current was 239.4A.

The specific loss power (SLP) is defined as the amount of electromagnetic energy lost per unit mass of magnetic material and is represented in watts per Gram (Wgm^−1^). In a magnetic hyperthermia experiment, it is proportional to the slope of the initial heating curve.

SLP is calculated in the following way for magnetic hyperthermia measurement (Kötitz et al., 1999),

$$\text{SLP}=\frac{cm_{S}}{m}\frac{dT}{dt}$$

Where, the specific heat of the solvent is denoted by c, the mass of the particle is denoted by m, the mass of the solvent is denoted by m_S_ and the slope of the linear region of the Temperature vs time curve is denoted by dT/dt.

SLP estimate is critical for determining the heating efficiency of magnetic composites and designing composites optimally in order to establish the effectiveness of magnetic hyperthermia.

**FIGURE 9:**
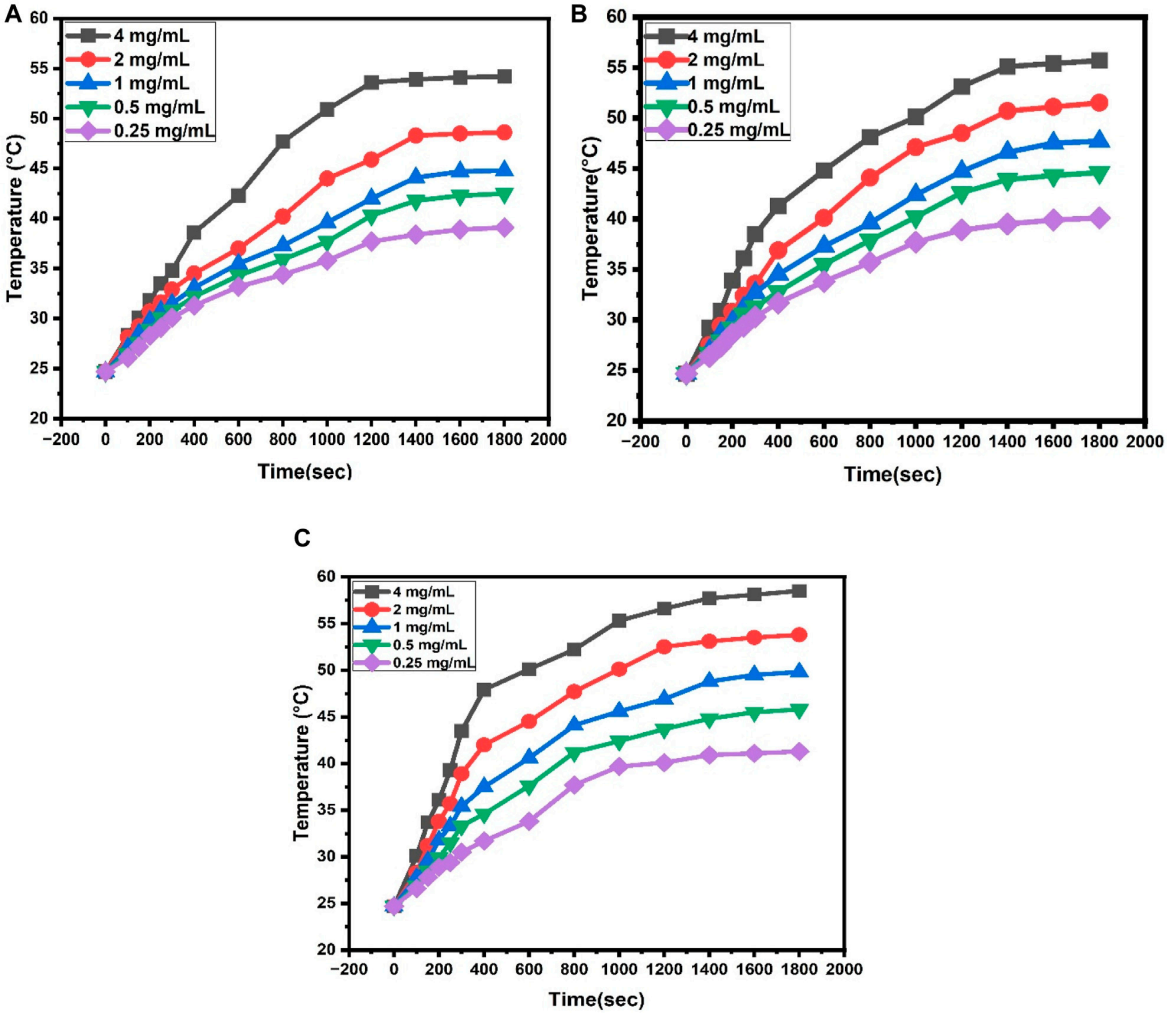
**(A)** Heating property of dextran-coated CaFe_12_O_19_/MnFe_2_O_4_ composites (calcination temperature 600°C) at different concentrations, **(B)** Heating property of dextran-coated CaFe_12_O_19_/MnFe_2_O_4_ composites (calcination temperature 800°C) at different concentrations, **(C)** Heating property of dextran coated CaFe_12_O_19_/MnFe_2_O_4_ composites (calcination temperature 1,000°C) at different concentrations.

The initial rise in the temperature with time was approximately linear. The Figure 9 illustrates the rate of heating increased with the concentration, as shown in the temperature vs time curves. To kill cancer cells, 42°C-46°C temperature is enough, and temperatures above 50 °C can cause damage to healthy cells (Baronzio and Hager, 2008).

**FIGURE 10:**
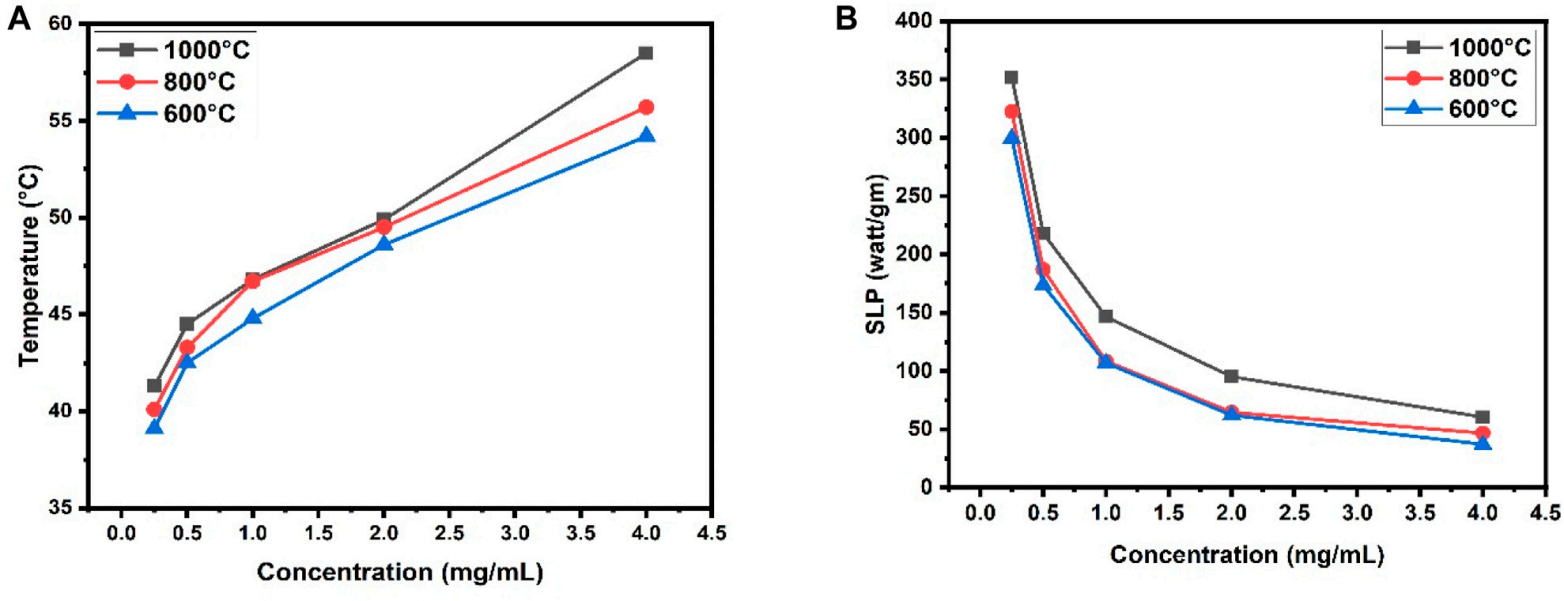
**(A)** Comparison among temperature vs. concentration calibration curve at three different calcination temperatures (600°C, 800°C, 1,000°C) of the prepared composite, **(B)** Comparison among SLP value against concentration at three different calcination temperatures (600°C, 800°C, 1000°C) of the prepared composite.

From the graphs (Figure 10), it can be observed that 0.5 mg/mL to 1.5 mg/mL concentration is optimal for hyperthermia treatment at calcination temperature 600°C. Similarly, for calcination temperatures 800°C, and 1,000°C optimal concentrations are respectively 0.5 mg/mL to 1.2 mg/mL, and 0.25 mg/mL to 0.5 mg/mL.

## 4 Conclusion

The heating profile shows that the necessary heat for the destruction of cancerous cells can be easily obtained from this material at three different calcination temperatures. The minimum concentration of foreign particle intake for hyperthermia is o.25 mg/mL for CaFe_12_O_19_/MnFe_2_O_4_ composite when calcined at 1,000°C.

## Data Availability

The original contributions presented in the study are included in the article/Supplementary Material, further inquiries can be directed to the corresponding author.

## References

[B1] Amighian J. Mozaffari M. Nasr B. (2006). Preparation of nano‐sized manganese ferrite (MnFe2O4) via coprecipitation method. Phys. status solidi C. 3 (9), 3188–3192. 10.1002/pssc.200567054

[B2] Baronzio G. F. Hager E. D. (2008). Hyperthermia in cancer treatment: a primer (Berlin, Germany: Springer Science and Business Media).

[B3] Dhiman R. L. Taneja S. P. Reddy V. R. (2008). Preparation and characterization of manganese ferrite aluminates. Adv. Condens. Matter Phys. 2008, 1–7. 10.1155/2008/703479

[B4] Dutz S. Hergt R. (2014). Magnetic particle hyperthermia—a promising tumour therapy? Nanotechnology 25 (45), 452001. 10.1088/0957-4484/25/45/452001 25337919

[B5] Garcia R. M. Bilovol V. Socolovsky L. M. (2012). Effect of the heat treatment conditions on the synthesis of Sr-hexaferrite. Phys. B Condens. Matter 407 (16), 3109–3112. 10.1016/j.physb.2011.12.038

[B6] Hoque S. M. Srivastava C. Kumar V. Venkatesh N. Das H. N. Saha D. K. (2013). Exchange-spring mechanism of soft and hard ferrite nanocomposites. Mater. Res. Bull. 48 (8), 2871–2877. 10.1016/j.materresbull.2013.04.009

[B7] Islam K. Haque M. Kumar A. Hoq A. Hyder F. Hoque S. M. (2020). Manganese ferrite nanoparticles (MnFe2O4): size dependence for hyperthermia and negative/positive contrast enhancement in MRI. Nanomaterials 10 (11), 2297. 10.3390/nano10112297 33233590 PMC7699708

[B8] Kim J. H. Hahn E. W. Ahmed S. A. (1982). Combination hyperthermia and radiation therapy for malignant melanoma. Cancer 50 (3), 478–482. 10.1002/1097-0142(19820801)50:3<478::aid-cncr2820500316>3.0.co;2-6 7093890

[B9] Kneller E. Khan Y. (1987). The phase Fe2B. Int. J. Mater. Res. 78 (12), 825–835. 10.1515/ijmr-1987-781201

[B10] Kötitz R. Weitschies W. Trahms L. Semmler W. (1999). Investigation of Brownian and Néel relaxation in magnetic fluids. J. magnetism magnetic Mater. 201 (1-3), 102–104. 10.1016/S0304-8853(99)00065-7

[B11] Lee J. H. Jang J. T. Choi J. S. Moon S. H. Noh S. H. Kim J. W. (2011). Exchange-coupled magnetic nanoparticles for efficient heat induction. Nat. Nanotechnol. 6 (7), 418–422. 10.1038/nnano.2011.95 21706024

[B12] Li T. J. Huang C. C. Ruan P. W. Chuang K. Y. Huang K. J. Shieh D. B. (2013). *In vivo* anti-cancer efficacy of magnetite nanocrystal-based system using locoregional hyperthermia combined with 5-fluorouracil chemotherapy. Biomaterials 34 (32), 7873–7883. 10.1016/j.biomaterials.2013.07.012 23876757

[B13] Pahwa C. Mahadevan S. Narang S. B. Sharma P. (2017). Structural, magnetic and microwave properties of exchange coupled and non-exchange coupled BaFe12O19/NiFe2O4 nanocomposites. J. Alloys Compd. 725, 1175–1181. 10.1016/j.jallcom.2017.07.220

[B14] Peiravi M. Eslami H. Ansari M. Zare-Zardini H. (2022). Magnetic hyperthermia: potentials and limitations. J. Indian Chem. Soc. 99 (1), 100269. 10.1016/j.jics.2021.100269

[B15] Predescu A. M. Matei E. Berbecaru A. C. Pantilimon C. Drăgan C. Vidu R. (2018). Synthesis and characterization of dextran-coated iron oxide nanoparticles. R. Soc. open Sci. 5 (3), 171525. 10.1098/rsos.171525 29657763 PMC5882687

[B16] Pullar R. C. (2012). Hexagonal ferrites: a review of the synthesis, properties and applications of hexaferrite ceramics. Prog. Mater. Sci. 57 (7), 1191–1334. 10.1016/j.pmatsci.2012.04.001

[B17] Remya K. P. Prabhu D. Amirthapandian S. Viswanathan C. Ponpandian N. (2016). Exchange spring magnetic behavior in BaFe12O19/Fe3O4 nanocomposites. J. Magnetism Magnetic Mater. 406, 233–238. 10.1016/j.jmmm.2016.01.024

[B18] Roy D. Kumar P. S. (2009). Enhancement of (BH) max in a hard-soft-ferrite nanocomposite using exchange spring mechanism. J. Appl. Phys. 106 (7). 10.1063/1.3213341

[B19] Shaterabadi Z. Nabiyouni G. Soleymani M. (2017). High impact of *in situ* dextran coating on biocompatibility, stability and magnetic properties of iron oxide nanoparticles. Mater. Sci. Eng. C 75, 947–956. 10.1016/j.msec.2017.02.143 28415550

[B20] Shinde V. S. Dahotre S. G. (2021). Comparative study of structural and magnetic properties of Ni and La substituted calcium hexaferrite. Cerâmica 67, 301–307. 10.1590/0366-69132021673833111

[B21] Shinde V. S. Dahotre S. G. Singh L. N. (2020). Synthesis and characterization of aluminium substituted calcium hexaferrite. Heliyon 6 (1), e03186. 10.1016/j.heliyon.2020.e03186 31989049 PMC6970159

[B22] Skomski R. Coey J. M. D. (1994). Exchange coupling and energy product in random two-phase aligned magnets. IEEE Trans. magnetics 30 (2), 607–609. 10.1109/20.312350

[B23] Song F. Shen X. Liu M. Xiang J. (2011). One-dimensional SrFe12O19/Ni0. 5Zn0. 5Fe2O4 composite ferrite nanofibers and enhancement magnetic property. J. Nanosci. Nanotechnol. 11 (8), 6979–6985. 10.1166/jnn.2011.4213 22103109

[B24] Tivol W. F. (2010). Selected area electron diffraction and its use in structure determination. Microsc. Today 18 (4), 22–28. 10.1017/S1551929510000441

[B25] Wolf P. (2008). Innovations in biological cancer therapy: a guide for cancer patients and their relatives. NaturaSanitas Publ.

[B26] Ye Z. Qie Y. Fan Z. Liu Y. Yang H. (2020). Exchange-coupled of soft and hard magnetic phases on the interfaces of Fe3C/CoFe2O4 nanocomposites. Ceram. Int. 46 (1), 731–736. 10.1016/j.ceramint.2019.09.026

[B27] Yu X. Ding S. Yang R. Wu C. Zhang W. (2021). Research progress on magnetic nanoparticles for magnetic induction hyperthermia of malignant tumor. Ceram. Int. 47 (5), 5909–5917. 10.1016/j.ceramint.2020.11.049

[B28] Zipare K. Dhumal J. Bandgar S. Mathe V. Shahane G. (2015). Superparamagnetic manganese ferrite nanoparticles: synthesis and magnetic properties. J. Nanosci. Nanoeng. 1 (3), 178–182.

